# Neuroanatomical correlates of working memory performance in Neurofibromatosis 1

**DOI:** 10.1093/texcom/tgac021

**Published:** 2022-05-19

**Authors:** Cameron Sawyer, Jonathan Green, Ben Lim, Gorana Pobric, JeYoung Jung, Grace Vassallo, D Gareth Evans, Charlotte J Stagg, Laura M Parkes, Stavros Stivaros, Nils Muhlert, Shruti Garg

**Affiliations:** Division of Neuroscience & Experimental Psychology, School of Biological Sciences, Faculty of Biology, Medicine and Health, University of Manchester, Oxford Road, Manchester M13 9PL, United Kingdom; Division of Neuroscience & Experimental Psychology, School of Biological Sciences, Faculty of Biology, Medicine and Health, University of Manchester, Oxford Road, Manchester M13 9PL, United Kingdom; Child & Adolescent Mental Health Department, Royal Manchester Children's Hospital, Central Manchester University Hospitals NHS Foundation Trust, Manchester Academic Health Sciences Centre, Manchester, Oxford Road, M13 9WL, United Kingdom; Child & Adolescent Mental Health Department, Royal Manchester Children's Hospital, Central Manchester University Hospitals NHS Foundation Trust, Manchester Academic Health Sciences Centre, Manchester, Oxford Road, M13 9WL, United Kingdom; Division of Neuroscience & Experimental Psychology, School of Biological Sciences, Faculty of Biology, Medicine and Health, University of Manchester, Oxford Road, Manchester M13 9PL, United Kingdom; School of Psychology, Precision Imaging Beacon, University of Nottingham, University Park, Nottingham NG7 2RD, United Kingdom; Manchester Centre for Genomic Medicine, Manchester University NHS Foundation Trust, Oxford Road, Manchester M13 9WL, United Kingdom; Manchester Centre for Genomic Medicine, Manchester University NHS Foundation Trust, Oxford Road, Manchester M13 9WL, United Kingdom; Division of Evolution and Genomic Sciences, Faculty of Biology, Medicine and Health, University of Manchester, Oxford Road, M13 9PL, United Kingdom; Wellcome Centre for Integrative Neuroimaging, Nuffield Department of Clinical Neurosciences & MRC Brain Network Dynamics Unit, University of Oxford, OX3 9DU, United Kingdom; Division of Neuroscience & Experimental Psychology, School of Biological Sciences, Faculty of Biology, Medicine and Health, University of Manchester, Oxford Road, Manchester M13 9PL, United Kingdom; Geoffrey Jefferson Brain Research Centre, Northern care Alliance NHS Foundation Trust, Stott Lane, Manchester M6 8HD, United Kingdom; Geoffrey Jefferson Brain Research Centre, Northern care Alliance NHS Foundation Trust, Stott Lane, Manchester M6 8HD, United Kingdom; Academic Unit of Paediatric Radiology, Royal Manchester Children's Hospital, Manchester University NHS Foundation Trust, Oxford Road, M13 9PL, United Kingdom; Division of Neuroscience & Experimental Psychology, School of Biological Sciences, Faculty of Biology, Medicine and Health, University of Manchester, Oxford Road, Manchester M13 9PL, United Kingdom; Division of Neuroscience & Experimental Psychology, School of Biological Sciences, Faculty of Biology, Medicine and Health, University of Manchester, Oxford Road, Manchester M13 9PL, United Kingdom; Child & Adolescent Mental Health Department, Royal Manchester Children's Hospital, Central Manchester University Hospitals NHS Foundation Trust, Manchester Academic Health Sciences Centre, Manchester, Oxford Road, M13 9WL, United Kingdom

**Keywords:** neuroimaging, NF1, VBM, working memory

## Abstract

**Introduction:**

Neurofibromatosis 1 (NF1) is a single-gene disorder associated with cognitive impairments, particularly with deficits in working memory. Prior research indicates that brain structure is affected in NF1, but it is unclear how these changes relate to aspects of cognition.

**Methods:**

29 adolescents aged 11-17 years were compared to age and sex-matched controls. NF1 subjects were assessed using detailed multimodal measurements of working memory at baseline followed by a 3T MR scan. A voxel-based morphometry approach was used to estimate the total and regional gray matter(GM) volumetric differences between the NF1 and control groups. The working memory metrics were subjected to a principal component analysis (PCA) approach.

**Results:**

The NF1 groups showed increased gray matter volumes in the thalamus, corpus striatum, dorsal midbrain and cerebellum bilaterally in the NF1 group as compared to controls. Principal component analysis on the working memory metrics in the NF1 group yielded three independent factors reflecting high memory load, low memory load and auditory working memory. Correlation analyses revealed that increased volume of posterior cingulate cortex, a key component of the default mode network (DMN) was significantly associated with poorer performance on low working memory load tasks.

**Conclusion:**

These results are consistent with prior work showing larger subcortical brain volumes in the NF1 cohort. The strong association between posterior cingulate cortex volume and performance on low memory load conditions supports hypotheses of deficient DMN structural development, which in turn may contribute to the cognitive impairments in NF1.

## Introduction

Neurofibromatosis 1 (NF1) is a common, single-gene autosomal dominant neurodevelopmental disorder with birth incidence of 1:2,700 (Evans et al. 2010). It is caused by mutations in tumor suppressor NF1 gene, which is crucial for regulation of the RasMAPK molecular pathway. NF1 affects multiple organs but mostly impacts the central nervous system (Daston and Ratner 1992). Consequently, it is associated with a range of cognitive impairments and is frequently comorbid with disorders such as attention-deficit hyperactivity disorder (ADHD) in ~40%–50% (Mautner et al. 2002; Garg et al. 2013) and autism spectrum disorder in 25% of the pediatric NF1 population. For a substantial majority, learning is affected (Hyman et al. 2005; Lehtonen et al. 2015) with impairments in all aspects of executive function (Plasschaert et al. 2016) including working memory impairments (Pobric et al. 2021).

The NF1 gene encodes for neurofibromin, which while ubiquitously expressed in the brain shows an enriched pattern of expression in the inhibitory interneurons (Ryu et al. 2019). Neurofibromin is critically involved in cell cycle regulation through negative regulation of the RasMAPK pathway. Studies in *Nf1* heterozygous knockout mice have shown that dysregulation of the RasMAPK pathway alters GABAergic neurotransmission both in the hippocampus and the cortex, with resultant deficits in synaptic plasticity and learning. GABAergic inhibition is important not only for the stability of neuronal networks, but it plays a fundamental role in shaping cortical development including regulating progenitor proliferation, migration, and maturation of neurons (Wang and Kriegstein 2009). In NF1, increased GABAergic activity disrupts plasticity during the critical periods (van Lier et al. 2020) and brain development. Studies in human clinical cohorts confirm GABAergic dysregulation but its link to cognition remains unclear (Violante et al. 2016). Macrocephaly early in development is one of the earliest and most well-replicated brain findings in individuals with NF1 (Baudou et al. 2019). Furthermore, T2-weighted hyperintensities (T2H) are commonly observed in the midbrain, brainstem, and thalamus, and more rarely in the deep cerebral white matter and cortex (DiPaolo et al. 1995; Roy et al. 2015) in 70% of the pediatric population (Sabol et al. 2011). They are thought to represent, in subcortical areas, foci of intramyelinic edema and in the deep white matter of the cerebrum and the cerebral cortex, areas of dysplasia, or hyperplasia (DiPaolo et al. 1995; Billiet et al. 2014) but their relationship to cognition is unclear (Torres Nupan et al. 2017). In particular, the presence, number, size, or location of T2H are not shown to be associated with executive function difficulties in NF1 (Roy et al. 2015).

Given both brain structure and function are affected in NF1, several studies have tried to link anatomical changes in the NF1 brain to aspects of cognition but with mixed results. A well-replicated finding is an increase in volume of the corpus callosum, which has been shown to be inversely proportional to verbal and nonverbal intelligence (Pride et al. 2010; Aydin et al. 2016) and to the severity of ADHD symptomatology (Kayl et al. 2000). Diffusion studies further indicate microstructural changes in the genu of the corpus callosum with a negative correlation between apparent diffusion coefficient (ADC) and arithmetic and digit span scores (Aydin et al. 2016). Increases in total brain volume, attributed to increases in either white matter or combined white and gray matter volumes (Payne et al. 2010; Karlsgodt et al. 2012), has not been found to be associated with neuropsychological function (Cutting et al. 2000). Studies of regional brain volume changes in NF1 have revealed hypertrophy in subcortical structures (such as the thalamus and putamen) and the cerebellum (Huijbregts et al. 2015) and less distinct gyrification particularly in the frontal and temporal regions (Violante et al. 2013). Huijbregts et al. (2015) found larger left putamen, right amygdala, and white matter volumes were associated with poorer executive function and autistic symptomatology in the NF1 population. Furthermore, studies indicate links between left/right asymmetry of the auditory cortex (planum temporale) and language ability and academic achievement (Barkovich et al. 2018). Associations between specific gray matter alterations and cognition have not yet been established.

In this study, we examined whether alterations in cortical and subcortical brain volumes in NF1 are linked to known cognitive deficits, particularly working memory. Working memory can be defined as short-term maintenance of information in the absence of sensory input. Working memory abilities play an important underlying role in acquisition of complex skills during development and strongly predict academic achievement (Gathercole and Alloway 2006). Along with the frontal lobes, the subcortical structures particularly the striatum support working memory function (Eriksson et al. 2015). Neurofibromin regulates prefrontal and striatal inhibitory networks and is required for working memory performance (Shilyansky et al. 2010). Several studies have demonstrated impaired working memory performance both in children and adults with NF1 (Plasschaert et al. 2016; Yoncheva et al. 2017; Tang et al. 2021). Furthermore, functional magnetic resonance imaging (fMRI) in NF1 clinical cohorts demonstrate differential activation of working memory networks (Ibrahim et al. 2017). Based on this evidence, our aim in this study was to investigate (i) differences in total and local brain volumes in the NF1 cohort as compared with typically developing controls and (ii) associations between regional gray matter volumes in NF1 and performance on working memory tasks. Based on the previous literature (Huijbregts et al. 2015), we predicted there would be hypertrophy within the subcortical structures particularly the lentiform nuclei.

## Methods

### Subjects

Thirty-one adolescents aged 11–17 years were recruited via the Manchester Centre for Genomic Medicine NF1 clinics and through NF charities newsletters and social media pages. Inclusion criteria included (i) clinical diagnosis made using the National Institute of Health diagnostic criteria (National Institutes of Health Consensus Development Conference 1988) and/or molecular diagnosis of NF1; (ii) no history of intracranial pathology other than asymptomatic optic pathway or other asymptomatic and untreated NF1-associated white matter lesion or glioma; (iii) no history of epilepsy or any major mental illness such as psychosis; and (iv) no MRI contraindications. Participants on pre-existing medications such as stimulants, melatonin, or selective serotonin re-uptake inhibitors were not excluded from participation. The sample size for the NF1 cohort was powered based on a larger intervention study in NF1 (Garg et al. 2021). All patients meeting the eligibility criteria for the study were sent study information packs in the post and were invited to return their indication of interest forms if they wished to participate in the study. Upon receiving the indication of interest form, the research team completed an eligibility questionnaire with the participants over the telephone to inquire about any NF1 associated difficulties.

Because of coronavirus disease of 2019 restrictions, it was not possible to collect study-specific control data. Instead, open-source healthy control images were used from OpenNeuro, a neuroimaging data-sharing platform (Stanford Center for Reproducible Neuroscience, United States, 2021). Two sets of control data were used in order to age-match with our NF1 participants: images from 13 individuals (sample HCa) were taken from a study by Padmanabhan et al. (2011) (age range = 11.2–17.5, mean age = 14.8, SD = 2.27, 6 females), and another 12 (sample HCb) were taken from a study by Geier et al. (2010) (age range = 11.7–18.5, mean age = 15.5, SD = 2.36, 5 females).

The patient and control groups were matched for age and sex (*P* = 0.45 and *P* = 0.29, respectfully). Critically there was also no significant difference in age (*P* = 0.45) or sex (*P* = 0.41) between the 2 control samples (HCa and HCb).

Two patient scans were excluded following quality checking due to significant artifacts, leaving 29 images of individuals with NF1 (15 female and 14 male) available for the study. All scans from the 25 age-matched HCs were included (11 female and 14 male).

### Standard protocol approvals, registrations, and patient consents

The study was conducted in accordance with local ethics committee approval (Ethics reference:18/NW/0762).

### Working memory assessments

For all NF1 participants detailed cognitive assessments were carried out to assess working memory. Verbal and visuospatial working memory was assessed using the *n*-back task. The task was programmed in-house using E-Prime software. Each participant completed verbal and visuospatial tasks at 4 levels of complexity—0-back, 1-back, 2-back, and 3-back tasks. For the verbal task, random letters were presented one at a time and the participant was asked to respond with a key-press if the letter corresponded to the letter 1 (1-back), 2 (2-back), or 3 (3-back) letters before. For the 0-back verbal task, participants were asked to press the key when they saw the letter “X.” For the visuospatial *n*-back task, blue squares were presented sequentially on a black 2 × 2 grid. Participants were instructed to respond with a key-press if the position of the square matched the position 1 (1-back), 2 (2-back), or 3 (3-back) positions before. For the 0-back visuospatial task, participants were asked to respond with a key-press when they saw an orange square. Each participant was presented with 3 blocks of each *n*-back task (24 blocks in total). All stimuli were presented for 500 ms and the interstimulus interval was set to 1,500 ms. Accuracy was calculated as the proportion of correctly identified hits + correct omissions within each block (correct hits + correct omissions/total responses) averaged across each *n*-back condition.

Memory span was assessed using a computerized Corsi block task on the Psychology Experiment Building Language (PEBL; Mueller and Piper 2014). In this task, 9 identical blue blocks are presented on the screen. These blocks light up on the screen in a sequence, which starts off as a simple sequence of 2 blocks and increases in complexity based on participant performance. The participant is asked to repeat the sequence observed on the screen. A measure of the memory span was computed. Auditory working memory was assessed using the digit span forward and back task of the Wechslers Intelligence Scale for Children-Fourth edition (WISC-IV; Goldstein and Naglieri 2011).

Parent-rated Vineland Adaptive Behavior Scale—third edition (Hill et al. 2017) was administered to the parents to assess child adaptive behavior with overall functioning computed as standardized age equivalent and expressed as an adaptive behavior composite (ABC). Conners 3 rating scale(Conners et al. 2011) was used as a standardized measure for parent reported ADHD symptoms. It consists of 27 items each rated on a 4-point Likert scale (0 = not true at all to 3 = very much true) in 5 subscales: attention, hyperactivity, learning problems, oppositionality, and peer problems. The inattention and hyperactivity subscales are reported below.

### Principal component analysis

Given detailed assessment of working memory, we used the principal component analyses (PCA) approach to boost statistical reliability by combining results from multiple tests and to avoid the problems of collinearity when undertaking brain region-symptom correlations. Such an approach has been used in previous stroke related neuroimaging studies (Halai et al. 2017). The working memory metrics including *n*-back task accuracy (0, 1, 2, and 3 back) on the verbal & visuospatial tasks, Corsi block memory span, and digit span forward and backward were entered into the PCA with varimax rotation. Factors with an eigenvalue > 1.0 were extracted and rotated. Following orthogonal rotation, the loadings of each test allowed a clear behavioral interpretation of each factor. Individual participant scores on each extracted factor were used as behavioral covariates in the neuroimaging analyses.

### MRI data acquisition and preprocessing

NF1 patient MRI data were acquired on a Philips Achieva 3T MRI scanner (Best, NL) using a 32-channel receive-only head coil. 3D T1-weighted MRI were acquired sagittally with a magnetization-prepared rapid acquisition gradient-echo sequence (MPRAGE; time repetition, TR = 8.4 ms; time echo, TE = 3.77 ms; flip angle = 8^o^, inversion time = 1,150 ms, 0.94 mm in-plane resolution and 150 slices of 1 mm).

Control data from HCa and HCb studies were acquired on a 3T Siemens Allegra scanner at Brain Research Centre, University of Pittsburgh. Structural data were acquired using sagittal 3D magnetization prepared rapid gradient-echo (MPRAGE; HCa (Padmanabhan et al. 2011): TR = 1.5 s, TE = 25 ms, flip angle = 70^o^, 224 slices of 0.7825 mm; HCb (Geier et al. 2010): TR = 1.5 s, TE = 25 ms, flip angle = 70^o^, 192 slices of 1 mm).

### VBM image processing

Images were segmented using SPM12 v7771 (Wellcome Trust Centre for Neuroimaging, University College London, United Kingdom) through MATLAB vR2020b (The MathWorks, Natick, MA, United States) into gray matter, white matter, and cerebrospinal fluid tissue classes using unified segmentation. We then used the diffeomorphic anatomical registration using exponentiated lie-algebra (DARTEL) registration method to normalize images and generate a unique gray matter template from the full study population (i.e. patients and controls). This nonlinear warping technique minimizes interindividual variation in neuroanatomy. All scans were checked following each stage of preprocessing for quality control. The final voxel resolution was 1.5 x 1.5 x 1.5 mm. Spatially normalized images were modulated by the determinants of the Jacobian so that voxel intensities represent the amount of deformation needed to normalize the images. Finally, all gray matter segmented images were smoothed with a 8-mm full-width half maximum isotropic Gaussian smoothing kernel.

### VBM statistical analysis

Voxel-based regression analysis (based on the GLM) was carried out using SPM12 using gray matter volume as the dependent variable. Age and sex were used as covariates of no interest (known to influence brain volume). Total intracranial volume was also calculated by summing the volumes of gray matter, white matter, and cerebrospinal fluid using the “get_totals” function in SPM12 and added as a global measure for proportional global scaling (in order to account for variation in head size) (Peelle et al. 2012).

For visualization purposes, all thresholded SPM maps were imported into MRIcroGL (https://www.nitrc.org/projects/mricrogl) and overlaid onto the automated anatomical labelling (AAL) atlas (Tzourio-Mazoyer et al. 2002).

Statistical analyses were carried out in the following stages:

1. We first analyzed group differences in regional gray matter volumes between the NF1 and healthy control participants, after accounting for age, sex, and total ICV.
2. Following from past literature, we tested the hypothesis that putamen and globus pallidus would show group differences in volume. We created a bilateral “lentiform nuclei” region-of-interest mask by combining left and right putamen and globus pallidus masks from the “Harvard-Oxford subcortical atlas.” We then used marsbar (Brett et al. 2002) to extract gray matter volumes within this region, which were compared between the NF1 and typically developing controls.
3. We then analyzed gray matter regions that show an association between increased volume and worse cognitive performance on each of the PCA factors in NF1 patients only, after adjusting for age, sex, and ICV.
4. We then looked at whether gray matter volumes within the “lentiform nuclei” mask from point 2 above were associated with PCA factors.

For points 1 and 3 above, we used family-wise error (FWE) of *P* = 0.05 to correct for multiple comparisons. For point 3 we also looked for any other regions throughout the brain that showed associations with cognitive performance at a more lenient threshold (*P* < 0.001, minimum cluster size = 10 voxels, uncorrected). For point 2, we assessed a single-group difference between patients and controls (*P* < 0.05), and for point 4, we controlled for the 3 PCA factor associations using Bonferroni correction (i.e. *P* < 0.017).

## Results

Clinical characteristics of the NF1 sample are presented in Table 1. There was a pre-existing diagnosis of ADHD in 8 participants and ASD in 3 participants. The overall Vineland adaptive functioning score was 68, well below the normative mean of 100. Parent-rated Conners-3 rating scale indicated significant impairments with T-scores of 78 and 69 on the inattention and hyperactivity subscales respectively.

**Table 1:** Clinical characteristics of the NF1 group.

Males	15/31
Age (mean)	14.7 yrs (11.4–18.3)
Pre-existing diagnoses (*n*)	
ADHD	8
ASD	3
Medications	
Stimulants	6
Atomoxetine	1
Vineland adaptive behavior composite (mean, SD)	68.4 (13.0)
Conners (mean, SD)	
Inattention *T* score	78.7 (13.0)
Hyperactivity *T* score	69.1(18.2)

**Fig. 1:**
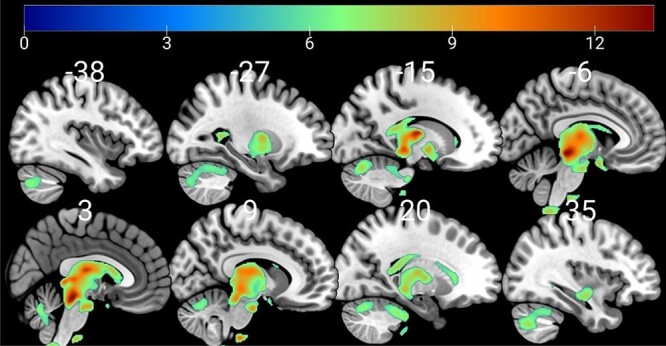
Sagittal sections showing regions of increased gray matter volume in the NF1 group compared with healthy control, superimposed on the MNI152 brain. Numbers above each slice represent MNI *x*- coordinates. Warmer colors represent regions of greater volume difference (see heat-map scale at top for equivalent *z*-values).

We first compared differences in gray matter volumes between the patients with NF1 and healthy controls. The NF1 group showed significantly larger volumes in 3 clusters—the first large cluster included a large portion of medial subcortical structures including the thalamus, globus pallidus, caudate, putamen, and dorsal midbrain, and cerebellum bilaterally (see Fig. 1), with the global maxima in left cerebellar lobules IV–V (*P* < 0.05 FWE corrected). There were 2 other smaller but still significant regions of volume change, with large NF1 volumes in the brain stem (937 voxels) and left caudate nucleus (63 voxels). In the region-of-interest analysis, NF1 patients showed significantly greater volume of the lentiform nuclei compared with healthy controls.

The working memory variables were subjected to a varimax rotated PCA, which produced 3 independent factors with eigenvalue > 1, which accounted for 54% of total variance as shown in Table 2. Measures of high working memory load loaded onto factor 1 (FAC1), low working memory load loaded onto factor 2 (FAC2), and auditory working memory on factor 3 (FAC3).

**Table 2:** Factor loading for PCA for working memory measures.

Factors	Factor 1	2	3
Verbal 0-back accuracy	0.340	**0.449**	0.427
Verbal 1-back accuracy	0.082	**0.808**	−0.009
Verbal 2-back accuracy	**0.563**	−0.001	0.332
Verbal 3-back accuracy	**0.848**	0.089	0.126
Visuospatial 0-back accuracy	0.065	**0.443**	0.454
Visuospatial 1-back accuracy	0.443	**0.643**	−0.012
Visuospatial 2-back accuracy	0.**790**	0.330	0.027
Visuospatial 3-back accuracy	**0.559**	0.201	0.238
Digit span forward	0.116	−0.063	**0.676**
Digit span backwards	0.212	0.071	**0.670**
Corsi block memory span	**0.488**	0.195	0.370

Highlighted factor loadings indicate which variables were used for the interpretation of the 3 factors.

**Fig. 2:**
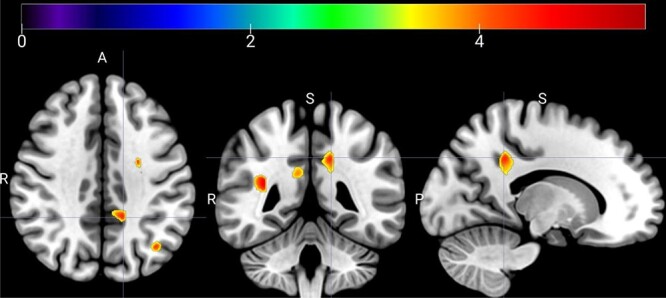
Multiplanar view of hypertrophic GM areas associated with worse low memory load capacity in adolescents with NF1. The crosshair is set at the largest voxel cluster at *x* = −15. Warmer colors represent regions with greatest associations between gray matter volume and FAC2 scores.

Next, we examined gray matter regions where volume was associated with performance on the cognitive tasks. For this, we used the first 3 principal components derived from the set of tasks, as described in the methods corresponding to high working memory load, low working memory load, and auditory working memory. Poorer performance on high working memory load tasks was significantly associated with increased volumes in a small region of inferior lateral parietal lobe (22 voxels). Poorer performance on low working memory load was significantly associated with increased volumes in a larger region of medial gray matter centered on the posterior cingulate cortex, alongside smaller regions of lateral parietal, middle temporal, and somatomotor regions (see Fig. 2 and Table 3). Auditory working memory performance was associated with gray matter volumes in a single smaller region centered on the middle frontal gyrus. Finally, the region-of-interest analysis revealed no significant correlation between lentiform nuclei volumes and either FAC1 (*r* = −0.01, *P* = 0.98), FAC2 (*r* = −.15, *P* = 0.45), or FAC3 scores (*r* = 0.26, *P* = 0.17). There were no significant associations between gray matter volumes and age in our NF1 sample.

**Table 3:** Neural correlates for PCA factors.

**Cognitive performance measure**	**Region of hypertrophy**	**Laterality**	**MNI coordinates**			**Voxel size (KE)**	***Z*-score (ZE)**
			***x***	***y***	***z***		
FAC1 (high memory load)	Inferior parietal lobe	L	−48	−51	60	22	3.29
FAC2 (low memory load)	Posterior cingulate gyrus	L	−15	−45	38	337	3.79
	Superior parietal gyrus	R	20	−69	57	238	4.41
	Precuneus	R	12	−50	26	211	3.69
	Middle temporal gyrus	R	35	−45	20	195	4.01
	Inferior precentral gyrus	L	−27	−3	41	165	3.99
	Supplementary motor area	R	9	−3	63	127	4.14
FAC3 (auditory)	Middle frontal gyrus	L	−24	26	20	132	4.18

**Fig. 3:**
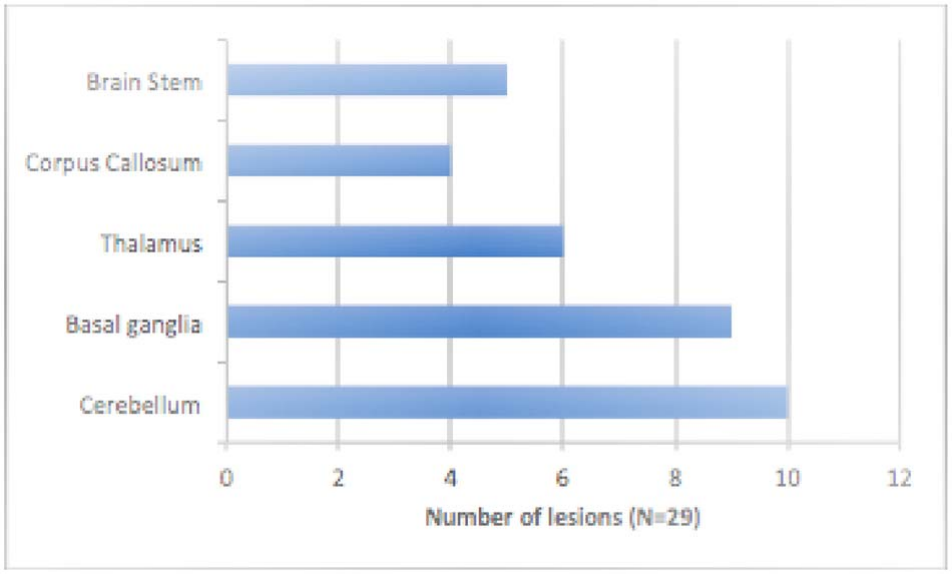
Graph showing the distribution of the T2 hyperintensities in the NF1 cohort.

T2H were present in 67% (20/30) of all NF1 participants in the study. The location of the T2 hyperintensities are shown in Fig. 3. The highest T2H were observed in the cerebellum, followed by the basal ganglia and the thalamus.

## Discussion

To our knowledge, this is the first study to investigate the neuroanatomical correlates of working memory performance in NF1. Results confirm previously reported findings (Huijbregts et al. 2015) of larger subcortical brain volumes in children with NF1 as compared with controls. These included the thalamus, globus pallidus, caudate, putamen, dorsal midbrain, cerebellum, and brainstem, which were larger in the NF1 group. Performance on the high load working memory tasks was inversely associated with volume of the inferior lateral parietal cortex. Low working memory load performance showed an inverse association with volume of posterior cingulate cortex, lateral parietal, middle temporal, and somatomotor regions.

Subcortical structures including the caudate, putamen, globus pallidus, and thalamus are part of fronto–striato–thalamo–cortical circuits that are essential for working memory and other higher executive functions including inhibition, attention and impulse control—all of which may be affected in NF1. Studies investigating the volumetric changes in the pediatric ADHD population have found significantly smaller volume of these subcortical structures (Nakao et al. 2011). Similarly, recent meta-analyses of morphometric studies in idiopathic ASD populations found smaller volumes of subcortical structures including the pallidum, putamen, amygdala, and nucleus accumbens (van Rooij et al. 2018). In contrast, we observe increases in the volumes of these subcortical structures in NF1. Given the important role for the NF1 gene in regulating cell growth and differentiation, the increased volumes may be a direct effect of gene expression (Ryu et al. 2019). Indeed, combined histological-MRI studies have shown that pathological changes in gray matter volume are linked to alterations in neuronal density and field fractions of glial markers (Eriksson et al. 2009). Volumetric changes in these regions thus may have important downstream effects on cognitive and social functioning impairments frequently seen in NF1 (Garg et al. 2013; Lehtonen et al. 2015). Volumetric changes in the cerebellum for instance, may underlie the previously reported deficits in fine and gross motor coordination, hypotonia and problems with balance reported in NF1 (Rietman et al. 2017). Moreover cerebellum was a commonly associated region for T2-hyperintensities observed in 67% of all participants, consistent with previous literature (Sabol et al. 2011). Future research should examine the longitudinal changes in the subcortical structures.

Brain regions showing significant association with working memory performance in this study are known to be key components of the default mode network (DMN). Specifically, increased volume of posterior cingulate cortex, lateral parietal, and middle temporal regions were associated with poorer performance on low working memory load conditions. Core “nodes” of the DMN include the posterior cingulate cortex, inferior parietal lobules, medial temporal lobe and the medial prefrontal cortex. Dynamic control of the DMN activity appears to be important for efficient cognitive function; fMRI studies show a pattern of increased brain activation at rest and reduced activation during attentionally demanding tasks in these DMN regions (Whitfield-Gabrieli and Ford 2012). Although fMRI studies in NF1 are limited, 2 previous task fMRI studies suggest possible failure of DMN deactivation during performance of a cognitive task, suggesting increased attentional lapses during performance of cognitive tasks may be reflected in neural activity within this region (Violante et al. 2012; Ibrahim et al. 2017). Resting state fMRI studies show reduced typical long-range connectivity between the posterior cingulate and medial prefrontal cortex- both hubs of the DMN (Chabernaud et al. 2012; Tomson et al. 2015). It is possible that the association between gray matter volumes in the DMN regions and cognition found in this study contribute to the DMN network abnormalities that in turn may underpin the cognitive impairments observed in NF1.

The posterior cingulate cortex is a highly connected, metabolically active brain region, known to be involved in self-referential processes including stimulus independent thought, day-dreaming, and autobiographic memory (Leech and Sharp 2014). The posterior cingulate is particularly relevant for dynamic reallocation of visuospatial attention-needed for working memory task performance (Leech and Sharp 2014). A previous fMRI study in an ADHD population noted reduced activation of the posterior prefrontal cortex during inhibition failure, suggestive of inefficient anticipatory attention reallocation (Rubia et al. 2005). Consistent with our results of inverse association of posterior cingulate cortex volume with working memory performance, studies in ADHD populations also show larger gray matter volumes of posterior cingulate cortex (Nakao et al. 2011). Taken together, posterior cingulate cortex appears to be an important DMN region mediating the NF1 associated risk of working memory impairments and ADHD symptomatology.

Our study is not without limitations. Due to a lack of local healthy control subjects, we used control data from openly available MRI datasets. We did however ensure that these datasets were matched for age and sex to our patients—in addition these data were only used for patient-control comparisons and the findings are in line with past work. Although these cohorts were scanned using different MRI machines, both patients and controls were acquired at 3T. Despite the variances this introduces, our findings are broadly in line with past literature and the brain volumes changes are marked, suggesting that they are not simply due to variances in structural scan sequence effects. As this work is cross-sectional, we cannot determine the ages at which hypertrophy is most accelerated. However, we found no associations between aging and increased volume in our NF1 sample, suggesting that increases may occur before adolescence. This would be useful to establish in future work, to understand the critical points of development for children with NF1. Future studies could combine VBM approach with functional approaches such as fMRI to better characterize brain structure–function relationship in NF1 (Mechelli et al. 2005). We did not exclude participants with comorbid diagnosis of ASD or ADHD. We acknowledge that stimulant medication could have a confounding effect on cognitive task performance. However in our sample, we found comparable performance between medicated and nonmedicated children with NF1 (data not shown). Given that ADHD and ASD affect 50% and 30% of patients with NF1 approximately, our sample can be considered representative of the NF1 population. Finally, regions with T2Hs were identified by colleagues in neuroradiology, but we did not use lesion outlining to map locations of lesions relative to areas of gray matter volume change. Future studies could examine whether these regions overlap or not, and what might drive these pathological effects.

Neuroimaging studies of the NF1 brain are important to understand the neural consequences of the NF1 mutations. As a single-gene disorder, NF1 present a valuable model to study the impact of the gene mutation on the brain and downstream on cognition and behavior. Our study elucidates the link between structural brain abnormalities in NF1 and working memory function. We confirm previous fundings of increased volume of subcortical structures and demonstrate a distinct neural signature associated with working memory impairments in NF1. Increased gray matter volumes of the DMN brain regions are associated with poorer working memory performance. Our findings support the hypothesis of deficient DMN structural development, which may in turn contribute to the cognitive impairments in NF1. These findings offer further insights into the brain basis of cognitive impairments seen in NF1.

Over the last decade, there have been several pharmacological interventions developed in NF1, targeting the underlying Ras-MAPK pathway activity. Preliminary evidence suggests that MEK inhibitors, used for the treatment of plexiform neurofibromas may also be effective for the treatment of working memory difficulties (Walsh et al. 2021). Further studies combining structural and functional brain imaging could be used to provide a deeper insight into neural mechanisms underlying cognitive functioning and inform strategies for future intervention trials.
